# Evaluation of hemodynamic effects of different inferior vena cava filter heads using computational fluid dynamics

**DOI:** 10.3389/fbioe.2022.1034120

**Published:** 2022-10-10

**Authors:** Mingrui Li, Jingying Wang, Wen Huang, Yue Zhou, Xue Song

**Affiliations:** ^1^ School of Energy and Power Engineering, Shandong University, Jinan, China; ^2^ The First Affiliated Hospital of Chongqing Medical University, Chongqing, China; ^3^ School of Aeronautical Science and Engineering, Beihang University, Beijing, China; ^4^ Jinan Central Hospital, Jinan, China

**Keywords:** hemodynamics, inferior vena cava filter, deep vein thrombosis, computational fluid dynamics (CFD), Denali filter

## Abstract

Inferior vena cava (IVC) filters are used to prevent pulmonary embolism in patients with deep vein thrombosis for whom anticoagulation is unresponsive. The head is a necessary structure for an Inferior vena cava filter (IVCF) in clinic use. At present, there are various head configurations for IVCFs. However, the effect of head pattern on the hemodynamics of IVCF is still a matter of unclear. In this study, computational fluid dynamics is used to simulate non-Newtonian blood flows around four IVCFs with different heads inside an IVC model, in which the Denali filter with a solid and hooked head is employed as a prototype, and three virtual variants are reconstructed either with a no-hook head or with a through-hole head for comparison. The simulation results show that the through-hole head can effectively avoid the recirculation region and weaken the blood flow stasis closely downstream the IVCF head. The shape change of the filter head has no significant effect on the blood flow acceleration inside the IVCF cone as well as little influence on the wall shear stress (WSS) distribution on the filter wire surface and IVC wall. The structure pattern of filter head greatly affects the flow resistance of its own. However, the flow drag of filter head only occupies a small proportion of the total resistance of IVCF. Therefore, to reduce the flow resistance of an IVCF should optimize its whole structure.

## 1 Introduction

Deep vein thrombosis (DVT) is a common vascular disease, preferably occurring in the lower extremities or pelvis (Heit, 2000). The thrombus in deep veins dislodges and flows toward the pulmonary artery, where larger emboli may cause pulmonary embolism (PE). PE is the most common complication of DVT with a high morbidity and mortality (Aycock et al., 2014; Chen et al., 2017). Both DVT and PE are collectively referred to as venous thromboembolism (VTE) (Yamashita et al., 2022). Currently, systemic anticoagulation is still the preferred method of prevention for VTE patients with underlying PE (Duffett and Carrier, 2017; Arslanbekov et al., 2021; Elias et al., 2021). Recent studies have shown that there is no additional benefit of IVCFs deployment in VTE patients with better anticoagulation therapy (Huang et al., 2021). The latest 2019 European Society of Cardiology (ESC) guidelines only recommend IVCFs for VTE patients who have absolute contraindications to anticoagulation or who still have PE recurrence after receiving anticoagulation (Konstantinides et al., 2020). For these groups, IVCFs have become an effective alternative to anticoagulant therapy. Although there have been some trials demonstrating that the IVCF is effective in preventing PE during and in the short term after trauma surgery (Kidane et al., 2012), no large randomized clinical trials demonstrate its long-term efficacy (Kaufman, 2018). Many studies also show that the use of IVCF can be accompanied by many complications, including filter displacement, rupture, and embolism of the heart and pulmonary circulation by filter fragments (Deso et al., 2016; Ahmed et al., 2019; Li et al., 2020). Retrievable IVCFs can either remain permanently in the patient’s blood vessels or be removed using percutaneous techniques after the patient’s PE risk has been reduced to a safe level. Retrievable IVCFs will become the main objective of filter improvement in the future (Ryan et al., 2017).

An ideal IVCF should be non-migratory and non-thrombogenic with high clot catching efficiency and vena cava patency. Actually, the IVCF configuration leads to the certain hemodynamics in IVC with the filter deployment, which affects and even determines the treatment effect of IVCF (Craven et al., 2018). Therefore, the structural optimization of IVCF is always a matter of concern. Recently, computational fluid dynamics (CFD) has become a useful and effective tool to study the effect of the filter structure on hemodynamic characteristics while both *in vivo* test and *in vitro* experiment have considerable difficulties of patient volunteers, setup incapability and high cost (Wang et al., 2019; Shar et al., 2020; Uchiyama et al., 2021; Yi et al., 2022).

Particularly, there are studies to suggest that the IVCF head has a specific impact on the hemodynamics inside the IVC with potential thrombosis. Singer et al. (2009) investigated the hemodynamic characteristics around TrapEase filter (Cordis, Miami Lakes, United States) using CFD methods. The results indicated that there is the obvious stagnant and recirculating flow downstream of the filter head, which possibly traps emboli for potential thrombogenesis. The simulation data of VenaTech convertible filter (B. Braun, Melsungen, Germany) by Wang et al. (2020) also showed the stagnation and recirculation zone downstream the filter head. At present, there are remarkable differences in the structure pattern of filter head between different IVCFs used in clinic. Some retrievable filter has the retrieval hook on the filter head, while some permanent IVCFs do not have. Additionally, the filter heads of several IVCFs are through-hole. Although the filter head is a necessary IVCF structure, the hemodynamic effects of its configuration are still short of study.

The objective of this paper is to evaluate the effects of the pattern of IVCF head on the hemodynamic characteristics inside the IVC with the filter deployment using CFD models. A retrievable Denali filter is employed as a basic prototype, and three virtual variants either with no-hook head or with hollow head are reconstructed on computer for comparison study. The blood flows around four filters with different heads in the IVC model are simulated in Ansys Fluent software (Ansys, Inc., Canonsburg, United States), which shows the distributions of blood velocity and WSS as well as the flow resistance on the blood flow exerted by each filter. By comparing the CFD results, the effects of different filter heads on the IVCF performance is discussed thoroughly.

## 2 Materials and methods

### 2.1 Geometric model

**FIGURE 1:**
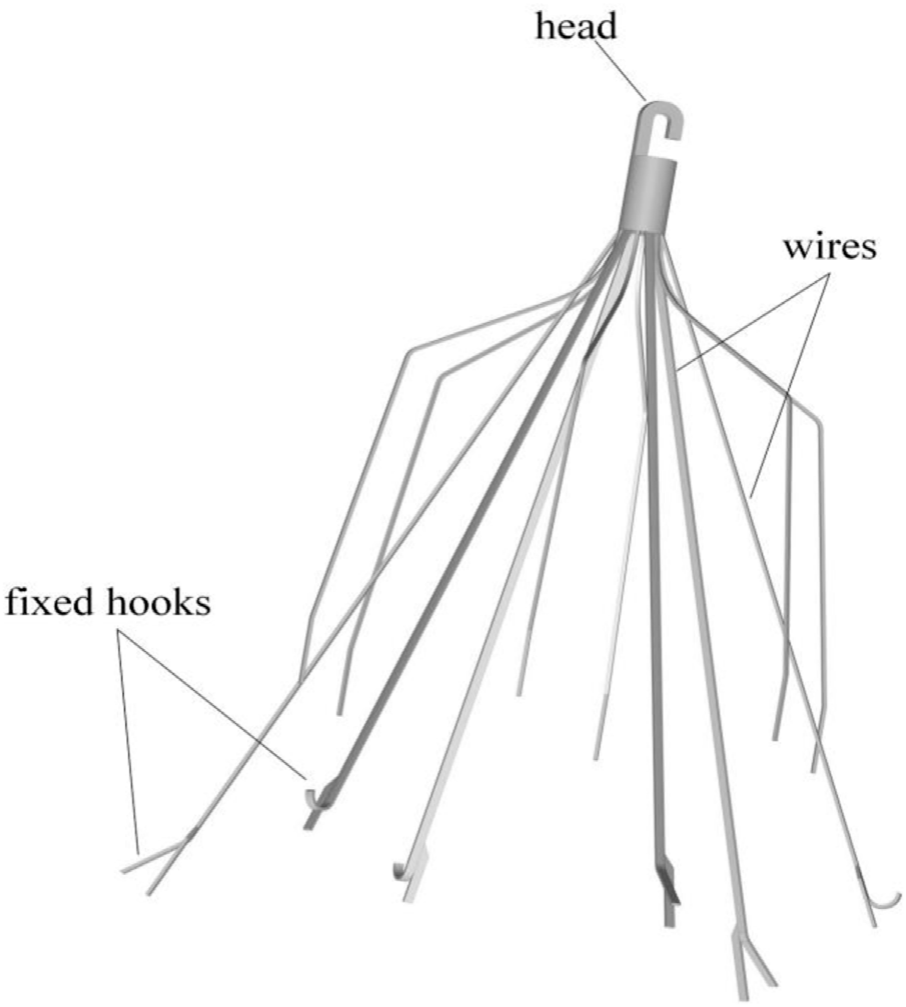
The Denali retrievable filter model.

The Denali vena cava filter (Bard Peripheral Vascular, Tempe, United States) is made of nickel-titanium alloy material, which received Food and Drug Administration (FDA) clearance in June 2010 (Hahn, 2015). As shown in Figure 1, the Denali filter has six long and short filtering wires to provide two levels of embolic filtration. The ends of the long wires have fixed hooks to prevent migration. The filter length is 50 mm, and has an indicated maximal filter diameter of 28 mm, per the manufacturer’s indications for use (Hahn, 2015). Unlike other retrievable IVCFs, Denali’s retrieval hook is cut from the same piece of nitinol, rather than welding a hook on the cylindrical head.

A baseline model is reconstructed on computer with the dimensions of a real Denali filter measured using a vernier caliper. Other three virtual filter variants are obtained on the basis of the basic model by only changing the filter head pattern either with no-hook or with through-hole. The geometries of all the four filter models are shown in Table 1. The four filter models remain in the state after deploying into the IVC, inside which they cannot be fully stretched. The height of the cylindrical filter head is equal to the maximum value of the length of the Denali filter head. And the outer diameter of the four filter heads and the inner diameter of the two hollow filter heads are equal. Table 1 numbers the four filters. When the IVCFs are deployed, since the fixed hooks are incorporated into the vessel wall in the actual treatment, the three-dimensional filter model actually used for CFD simulations ignores this structure.

**TABLE 1:** Four filter models for simulation.

Label	A	B	C	D
Front view	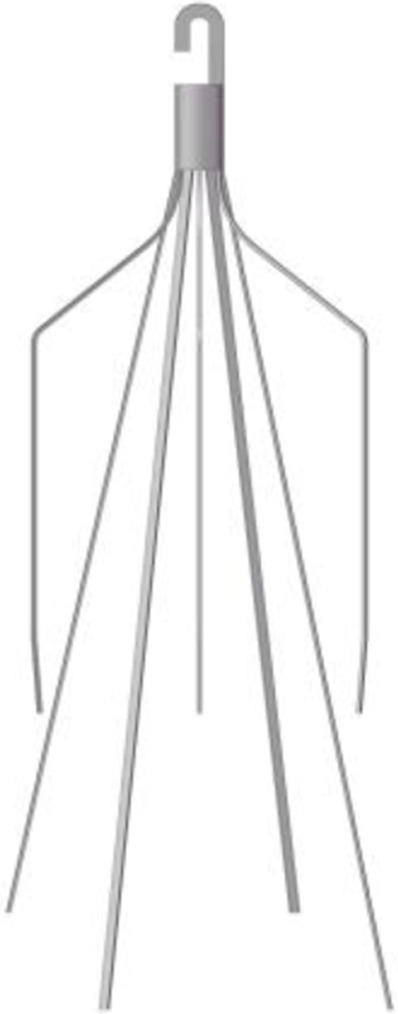	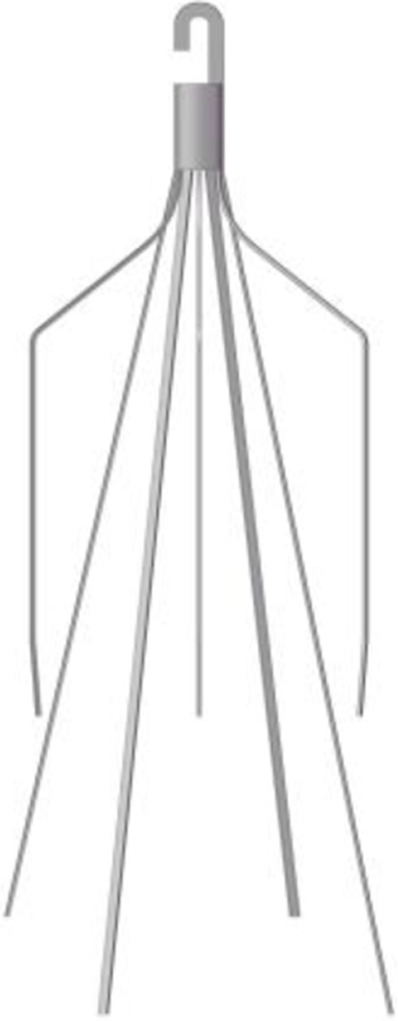	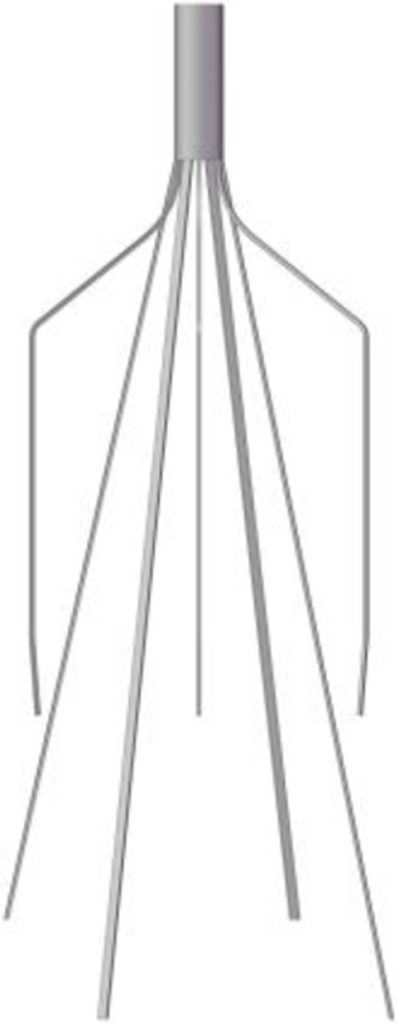	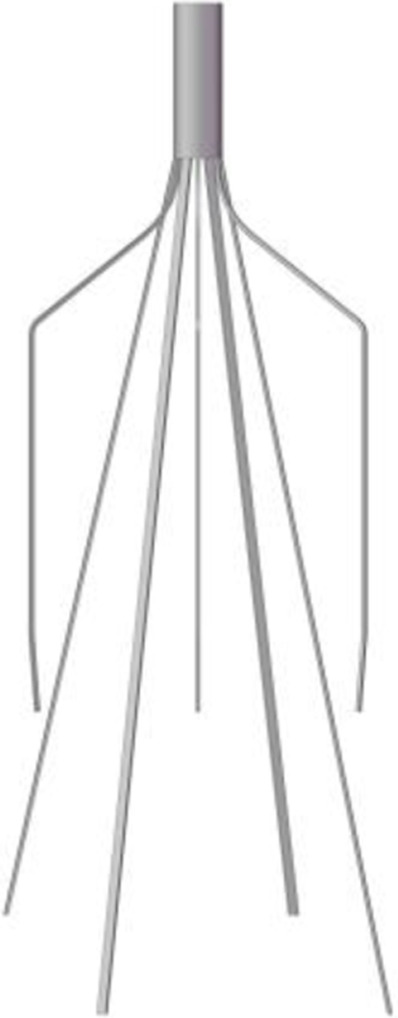
Filter head pattern	Basic	Hollow	Solid cylinder	Hollow cylinder

For simplicity, three-dimensional model of the IVC is constructed as a rigid cylindrical tube with a diameter (*D*) of 20 mm as many previous studies have done. Flow extensions are added at the inlet and outlet sections, respectively, the lengths of which are both nearly 20 times *D* to ensure fully developed laminar flow and avoid the unreasonable boundary effects of simulation (Wang et al., 2020).

### 2.2 Mathematical methods and boundary conditions

Blood is a non-Newtonian fluid with shear-thinning properties. It is generally accepted that the Newtonian model can be used in place of the non-Newtonian model when describing blood flow in arteries (Perktold et al., 1989). However, the Newtonian model cannot accurately predict hemodynamics, especially for WSS, when simulating the hemodynamic characteristics in IVC (Aycock et al., 2016). In this study, the Carreau model is used to describe the non-linear relationship between blood viscosity *μ* and shear rate *γ* as follows (Cho and Kensey, 1991):

$$\mu =\mu_{\infty }+\left(\mu_{0}-\mu_{\infty }\right){\left[1+{\left(\lambda \gamma \right)}^{2}\right]}^{\frac{n-1}{2}}$$ (1)

where *n* = 0.3568, *λ* = 3.313 s, *μ*_*∞*_ = 0.00345 Pa·s and *μ*_*0*_ = 0.056 Pa·s. These parameter settings are matched with human blood (Tabakova et al., 2014). The Carreau model has been widely used and validated to be adequately accurate for characterizing the non-Newtonian properties of blood.

This work focuses on comparing the blood flow features around four filters. Some complicated physiological factors (such as the IVC elasticity (Tedaldi et al., 2018), the approximately IVC elliptical structure, and the eventual side branches (Rahbar et al., 2011), etc.) are not be considered in this paper. It reasonably assumes the IVC blood flow is steady due to its low pulsation and low pressure measured in the clinic (Leask et al., 2004). Therefore, the IVC blood flow over the filter can be characterized by the steady, viscous and incompressible Navier–Stokes (N–S) equations (Tian et al., 2013; Liu et al., 2018) as follows:

∇⋅V=0 (2)

$$\rho V\cdot \nabla V=-\nabla p+\mu \nabla^{2}V$$ (3)

where “∇” is the gradient operator, ***V*** is the velocity vector of blood flow, *ρ* is the blood density, and *p* is the pressure.

**FIGURE 2:**
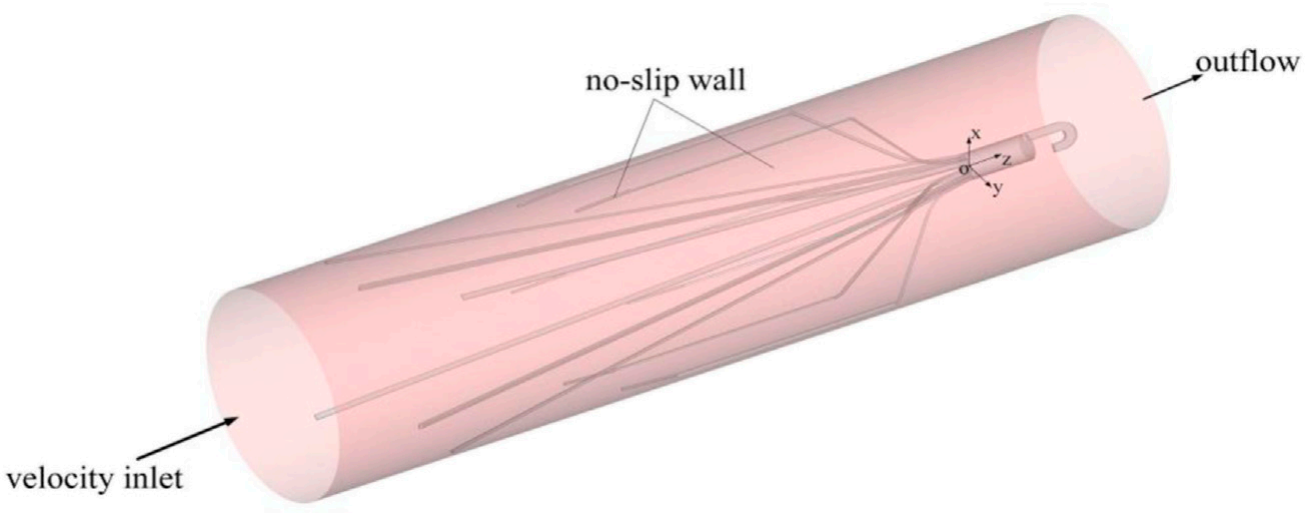
Filter in an IVC model and boundary conditions.

The setting of boundary conditions directly affects the accuracy of simulation results. Figure 2 is a sketch of the Denali filter deployed inside the IVC model for the present CFD simulation, and shows the boundary conditions. One end of the IVC tube is regarded as an inlet, while the other end is considered as an outlet. At the IVC inlet, the mean flow speed (*V*_*m*_) is set to be 0.07 m/s (corresponding to a volume flow of about 1.36 l/min). This speed is within the range of human inferior vena cava blood velocity measured *in vivo* (Cheng et al., 2003). *ρ* is set as 1,060 kg/m^3^ (López et al., 2018). The surfaces of the filters and IVC tube are both set as no-slip walls where the blood flow speed is zero. Eqs 1–3 with all boundary conditions are solved in the software Ansys Fluent for the present CFD simulation.

The Reynolds number, Re, is calculated as follows:

$$Re=\frac{\rho V_{m}D}{\mu_{a}}$$ (4)

where *μ*_*a*_ is the spatially averaged viscosity. According to the following CFD results, the spatially averaged viscosity for all four cases is about 0.0058 Pa·s, and the corresponding Re is 263, which determines that the blood flow in the present study is laminar.

## 3 Verification and validation

### 3.1 Grid refinement study

**FIGURE 3:**
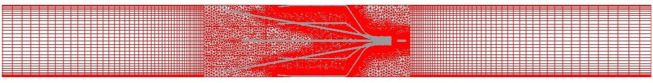
CFD grids.

The unstructured meshes are created in the vessel containing the IVCF model, and the structured grids are generated for the upstream and downstream extension connected to the IVCF segment, as shown in Figure 3. The blood flow mainly changes varies around the filters and near the vessel wall, where the meshes are locally refined.

**FIGURE 4:**
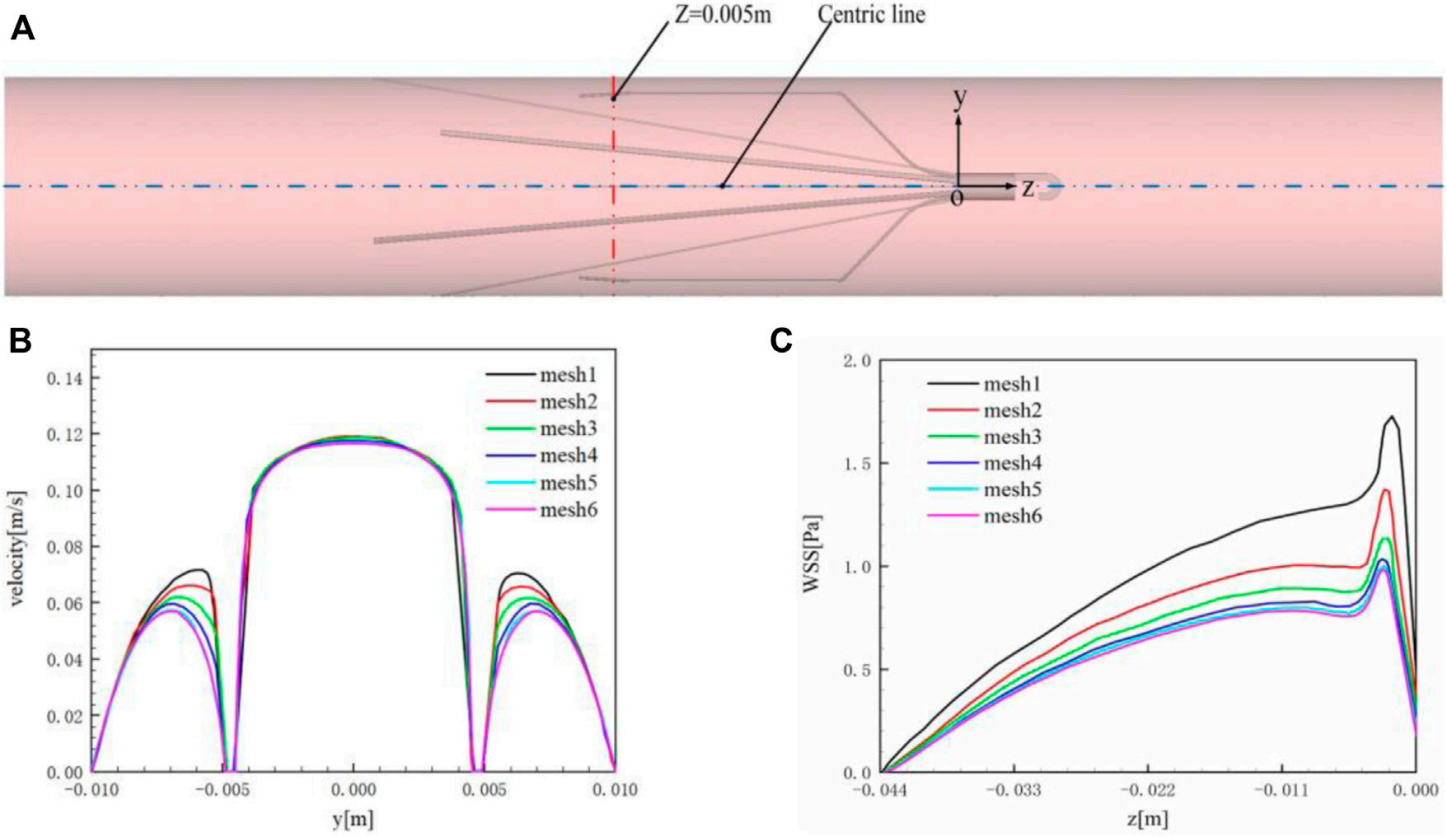
**(A)** *z* = 0.005 m cross-section and centric line (*x* = 0 m, *y* = 0 m); **(B)** velocity profile in the *y*-axis direction at *z* = 0.005 m cross-section; **(C)** *WSS*_*avg*_ of the filter surface.

The main purpose of grid verification is to reduce the discrete error as much as possible on the premise of saving computing resources. Therefore, the present grid verification mainly consists of two parts: 1) qualitatively analyze the influence of the total number of grids on the calculation results; 2) use the grid convergence index (GCI) to quantitatively evaluate the numerical precision of the target parameters (Yi et al., 2022). A total of six meshes list in Table 2 are used to simulate the Denali filter case for grid verification. For 1), the velocity profile in the *y*-axis direction at the *z* = 0.005 m cross-section and the WSS distribution along the long wire of Denali filter are compared. Figure 4A shows the *z* = 0.005 m cross-section and the centric line (*x* = 0 m, *y* = 0 m) of the IVC model. Figures 4B,C show that with the increase of total number of grids from mesh 1 to 6, the velocity and WSS lines gradually reach unchanged. For 2), the maximum velocity magnitude (*U*_*max*_) on the centric line and the area-weighted average WSS (*WSS*_*avg*_) at the filter surface are used as the target parameters to calculate the convergence orders *ψ* and GCI for mesh 3 to 6, respectively. In Table 2 *WSS*_*avg*_ is formulated as follows (Wang et al., 2020):

$${WSS}_{avg}=\frac{1}{\varphi }\int_{\varphi }WSS\cdot d\varphi$$ (5)

where *φ* is the surface area of the Denali filter. As the total number of grids increases, the GCIs show a decreasing trend from mesh 3 to 6. It indicates that *U*_*max*_ and *WSS*_*avg*_ gradually get convergent with the grid refinement. The GCIs of *U*_*max*_ and *WSS*_*avg*_ for mesh 6 are 0.40% and 0.23%, respectively, which indicates that the numerical solution of mesh 6 is adequately precise. Therefore, the total number of grids for all cases in the current study is set to be 3.6 million.

**TABLE 2:** Quantitative analysis of CFD grid uncertainty.

Total number of grids		*U*_*max*_ (m/s)	*ψ*	GCI(%)	WSS_*avg*_ (Pa)	*ψ*	GCI(%)
Mesh 1	4.91E+05	0.093			1.042		
Mesh 2	7.53E+05	0.096			1.072		
Mesh 3	1.09E+06	0.114	2.80	4.55	1.088	4.80	2.10
Mesh 4	1.63E+06	0.122	4.39	2.30	1.097	4.39	1.31
Mesh 5	2.44E+06	0.125	7.49	1.70	1.101	6.19	0.36
Mesh 6	3.73E+06	0.126	8.38	0.40	1.103	5.29	0.23

### 3.2 Experiment validation

Although the suitable mesh is used, it still needs to validate the solution of the present CFD method is reliable compared with the experimental or clinical data. Due to the lack of experimental and clinical data for the Denali filter, this work uses the *in vitro* measurement data of the TrapEase filter deployed in a rigid IVC model for CFD validation (Leask et al., 2004).

**FIGURE 5:**
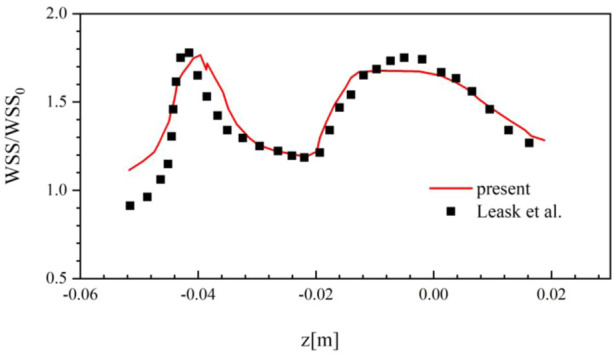
Comparison of the simulation results and the experimental results.

In the TrapEase filter experiment, the inlet flow rate is set to be 17.8 ml/s, and the density of blood substitute is 0.756 g/cm^3^ with the constant dynamic viscosity of 0.00143 Pa·s. Figure 5 compares the WSS results at the surface of the IVC model around the TrapEase filter predicted by the present CFD method with those measured in the experiment, where WSS is non-dimensionalized by the theoretical wall shear stress *WSS*_*0*_ (0.032 Pa) for a Poiseuille flow in a long straight pipe. It can be found that the current CFD results agree well with the experimental results. The overall relative average difference between the two sets of data is within 15%, which very possibly derives from the error of the three-dimensional model reconstruction of the TrapEase filter. In general, the present CFD solver is reliable for simulating the steady laminar flow of blood in a circular straight tube with an IVCF deployment.

## 4 Results

### 4.1 Blood flow velocity

**FIGURE 6:**
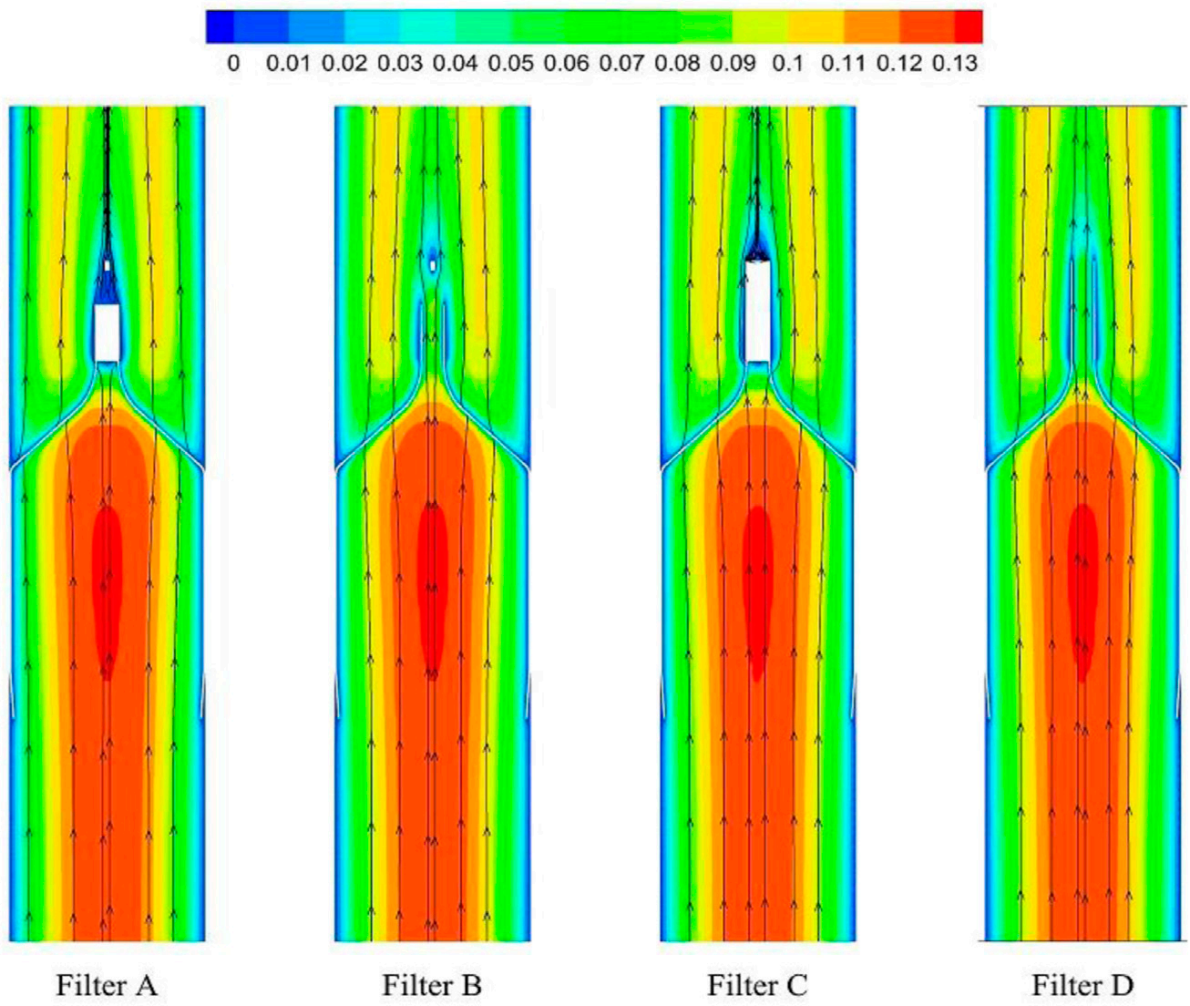
Blood flow velocity distribution in axial section.

**FIGURE 7:**
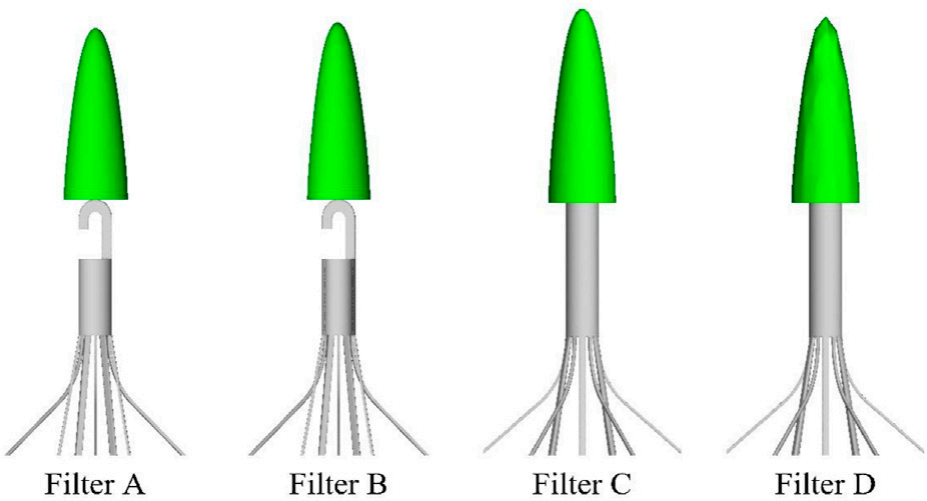
Three-dimensional blood flow velocity isosurface (*V* = 0.8*V*_*m*_ = 0.056 m/s).

As demonstrated in Figure 6, the stagnation zones can be observed downstream from the filter heads. There are the recirculation regions just downstream the solid filter heads (Filters A and C), while none from the hollow filter heads (Filter B and D). The filter with hollow head has the flow with higher speed downstream the filter head than the filter with solid head. Defining the present stagnation zone is the region where the magnitude of velocity is less than 0.8 times the mean blood flow velociry, *V*_*m*_. In Figure 7, a velocity isosurface (*V* = 0.8*V*_*m*_ = 0.056 m/s) shows the volume of three-dimensional stagnation zone for each case. It shows that the stagnation effects of the Filters C and *D* are more serious than Filters A and B.

**FIGURE 8:**
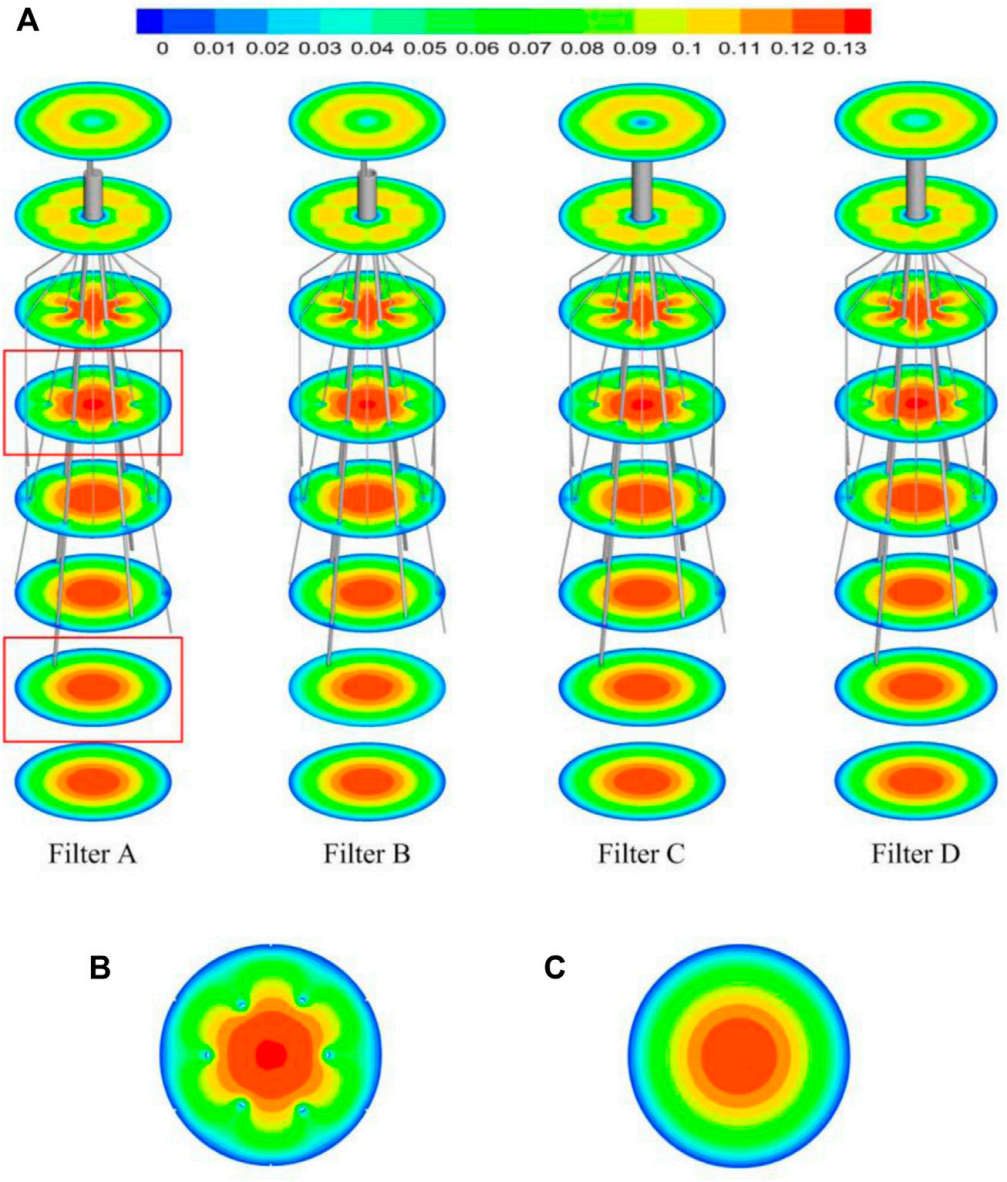
**(A)** The velocity distribution in the cross section; **(B)** the velocity distribution in the cross section affected by the filter; **(C)** the velocity distribution in the cross section not affected by the filter.

**FIGURE 9:**
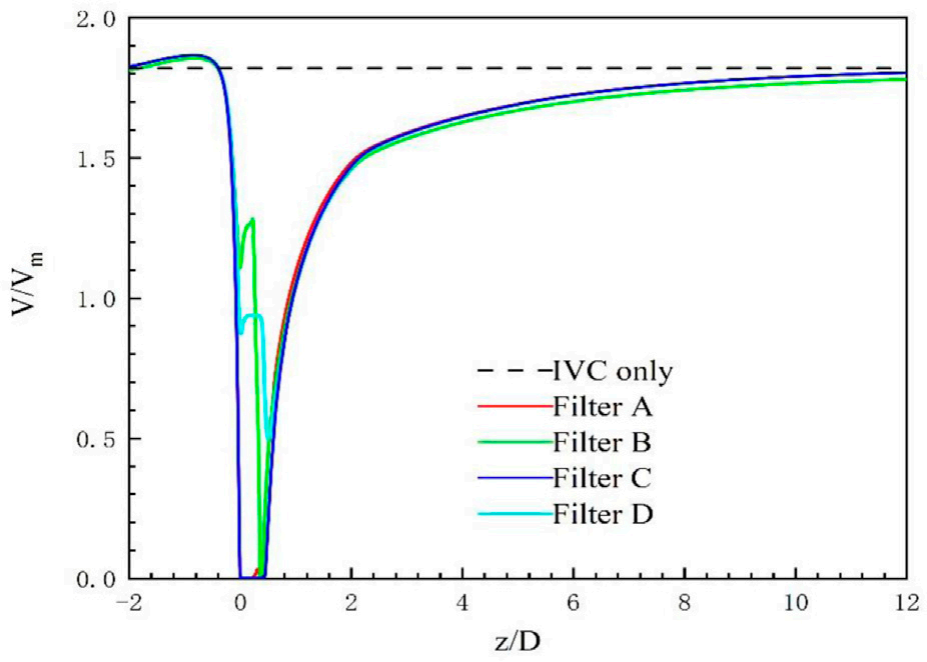
Blood flow velocity on the central line of the five cases.

The high-speed regions are clearly observed inside the filter cone as well as between two arbitrary filter wires for all the cases, as shown in Figure 8A. Furthermore, Figure 8B shows that there is a significant acceleration effect in the center of the cone compared to the case without the filter deployment as depicted in Figure 8B. In order to investigate the influence of the filter head shape on the acceleration effect, the variations of blood flow velocity along the IVC centric line for four filters with different heads are plotted in Figure 9, where the data of the case of the IVC with no filter deployment is used as the baseline. The velocity in Figure 9 is non-dimensionalized by *V*_*m*_, and the *z*-ordinate is normalized by *D*. It can be found that changing the shape of the filter head has almost no significant impact on the acceleration effect inside the filter cone. The maximum values of blood flow velocity for Filters A–D increase by a factor of 5.24%, 4.17%, 4.19%, and 4.23% of Vm, respectively, compared to the baseline case without the filter.

### 4.2 Wall shear stress

**FIGURE 10:**
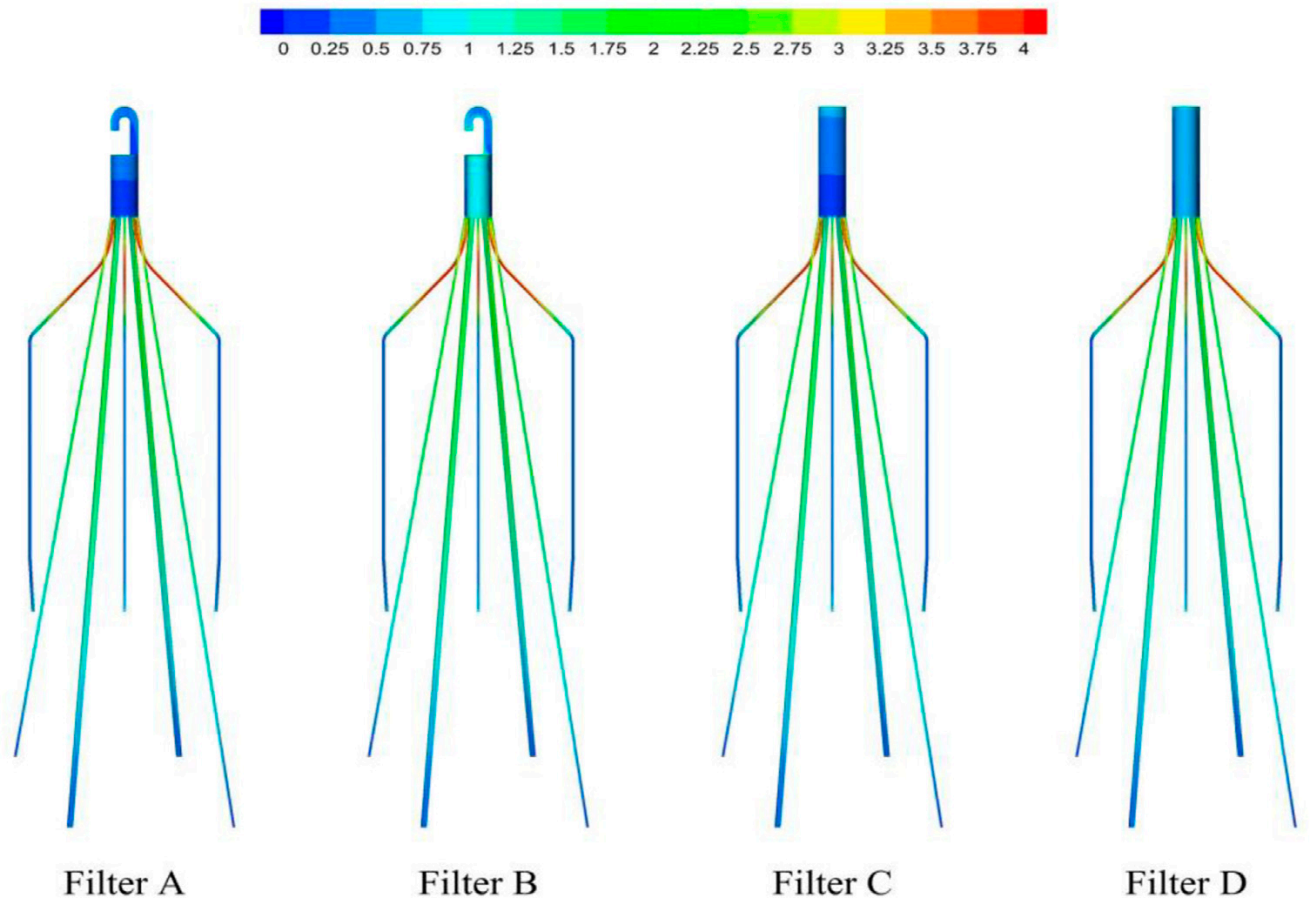
WSS distribution on filter surface.

**FIGURE 11:**
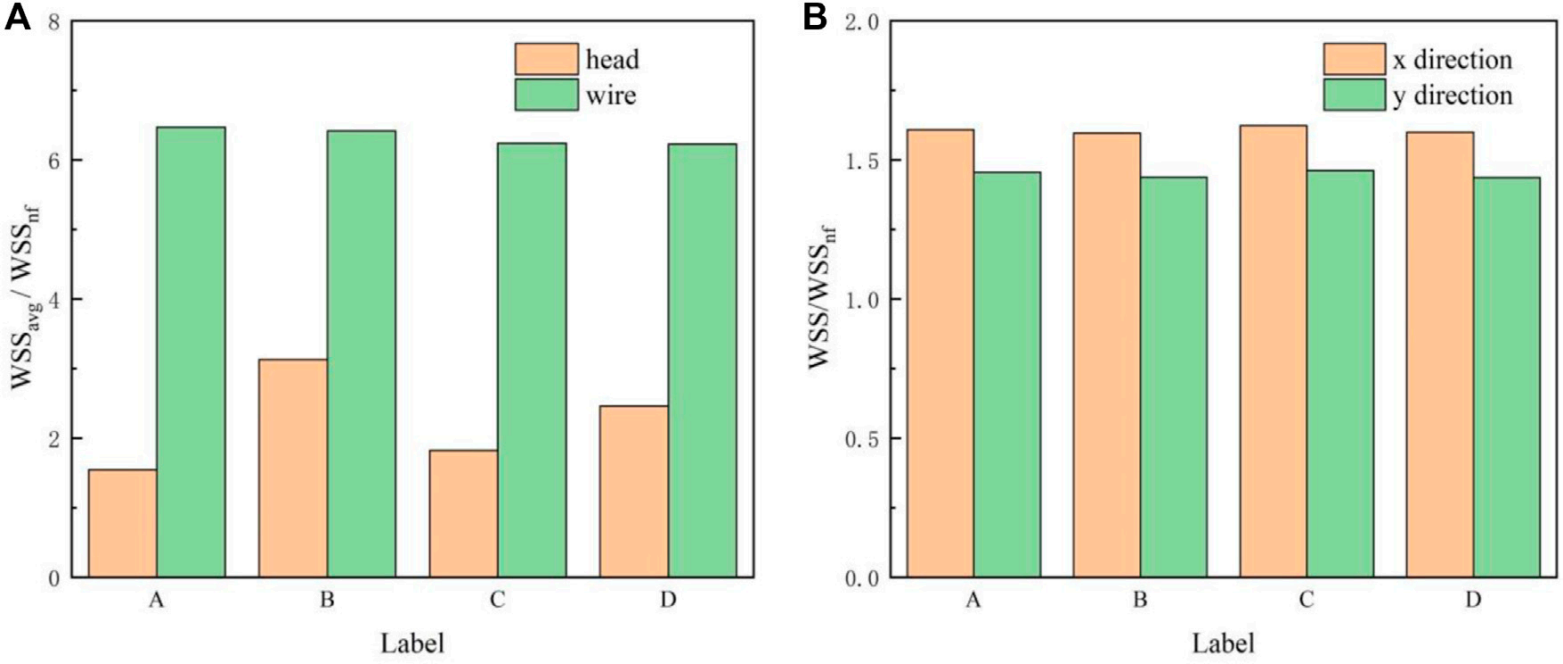
**(A)** *WSS*_*avg*_ on the filter head or wire surface; **(B)** *WSS*_*max*_ on the IVC surface in the *x* and *y* directions.

Generally, due to the acceleration of blood flow around the filter wire, the WSS on the upstream side of the filter wire (*WSS*_*wire*_) is much higher than that on the downstream side. *WSS*_*wire*_ increases continuously, reaches a maximum near the filter head, and finally decreases rapidly in a short distance, as shown in Figure 10. The distribution of *WSS*_*wire*_ on the four filter surfaces is not significantly different. It is worth noting in Figure 11A that the *WSS*_*avg*_ on the filter surface is 6 times that of WSS_*nf*_, which is the WSS value of the IVC with no filter implant. The vertical axis in Figure 11 is non-dimensionalized by *WSS*_*nf*_ = 0.1908 Pa. The WSS distribution on the filter head (*WSS*_*head*_) is obviously different. Figure 11A shows that for the same filter head pattern (with or without hook), the hollow one leads to an increase in *WSS*_*avg*_ on the filter head surface. The *WSS*_*avg*_ on the filter head surface of Filter B is the largest, which is twice as large as that of Filter A. The *WSS*_*avg*_ on the filter head surface of Filter D is also much larger than that of Filter C.

**FIGURE 12:**
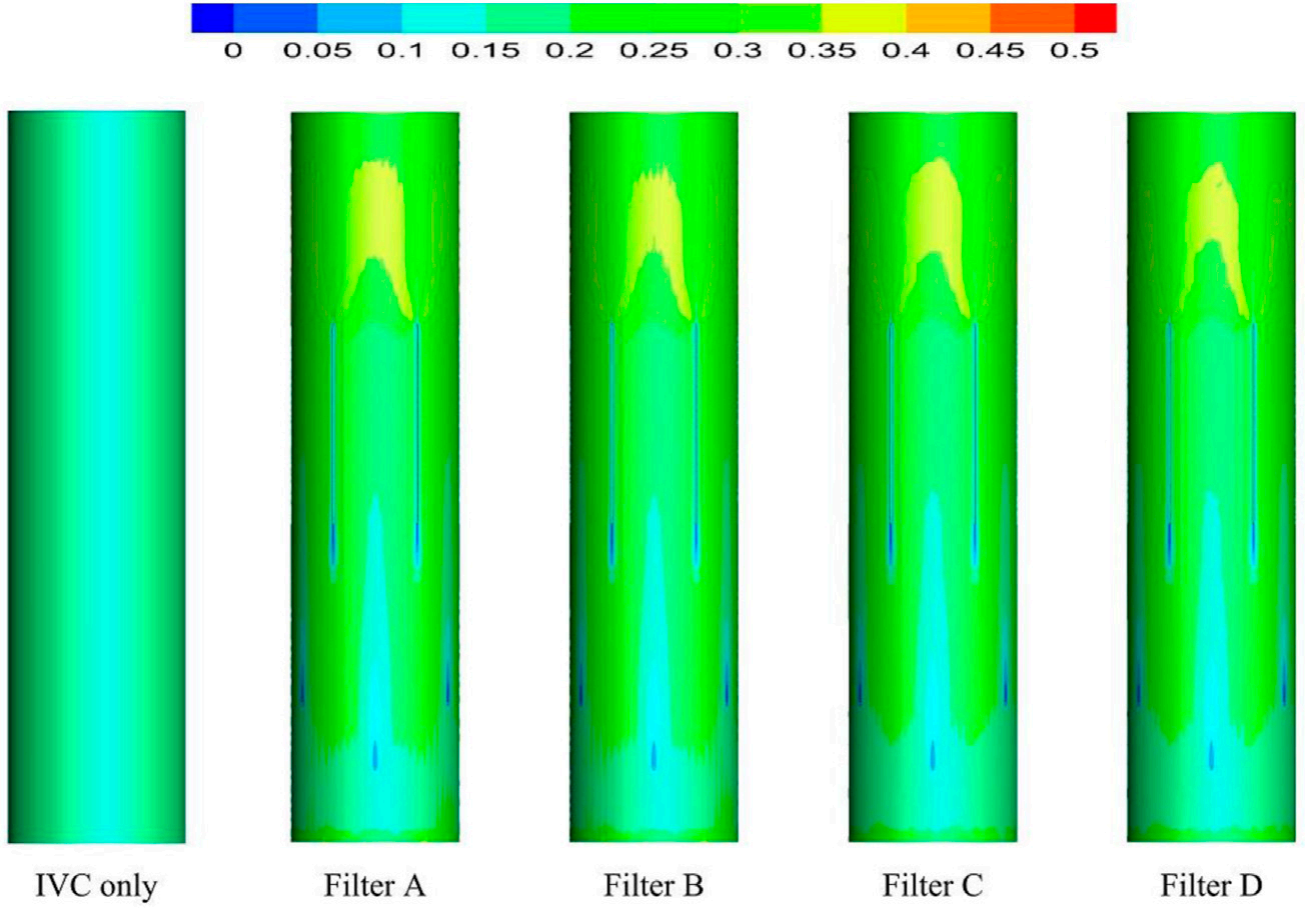
WSS distribution on IVC surface.

Deployment of the filter severely altered the WSS distribution of the IVC part surrounding the filter (*WSS*_*IVC*_), as shown in Figure 12. The *WSS*_*IVC*_ is smaller near the positions where the filter wires are in contact with the IVC. However, there is no significant difference in the distribution of *WSS*_*IVC*_ for four cases. Figure 11B shows that maximum values of the WSS around the four filter heads (*WSS*_*max*_) are also very close.

### 4.3 Flow resistance

The flow resistance (*F*_*f*_) exerted by the blood flow on the filter consists of a viscous drag (*F*_*v*_) and a pressure drag (*F*_*p*_). Table 3 lists the viscous drag, pressure drag and flow resistance of the filter wire and head for all the four filters. The total flow resistances (*F*_*f*_) of four filters have little difference. For all the four filters, the viscous drag (*F*_*v*_) makes much contribution to flow resistance (*F*_*f*_) than the pressure drag (*F*_*p*_). For each case, both the viscous drag and pressure drag of the filter wire are much greater than those of the filter head, which means the filter wire is the main contributor of the blood flow resistance of an IVCF. It is noticeable that the viscous drag of the filter head of Filter D is obviously greater than that of any other filter, while the pressure drag of the filter head of Filter D is far smaller than that of any other case.

**TABLE 3:** Viscous drag, pressure drag and flow resistance for four filters with different heads (unit: dyne).

Force	A			B			C			D		
	Wire	Head	Total	Wire	Head	Total	Wire	Head	Total	Wire	Head	Total
*F*_*v*_	55.0	1.2	56.2	56.0	1.5	57.5	54.9	2.0	56.9	53.9	5.4	59.3
*F*_*p*_	15.2	5.2	20.4	14.9	4.4	19.3	15.1	5.3	20.4	14.4	1.1	15.5
*F*_*f*_	70.2	6.4	76.6	70.9	5.9	76.8	70.0	7.3	77.3	68.3	6.5	74.8

## 5 Discussion

The head is a necessary part of the IVCF for use. The pattern of the filter head has an important effect on the IVCF hemodynamics. In this paper, the Denali filter is employed as a baseline model, the head of which is virtually changed into three other different patterns. Then, CFD techniques are used to simulate the blood flow around the four filters with different heads. The hemodynamic characteristics of the four filters are all discussed in detail including the blood flow velocity profiles, WSS distributions and the flow resistance. To our knowledge, the present comparison study has not been shown in previous experimental or computational studies.

### 5.1 Stagnation zones and recirculation regions

Wang and Singer (2010) used CFD to simulate the hemodynamic characteristics of TrapEase and Celect filters deployed into the averaged IVC models, respectively. Their IVC model included the left and right renal veins but ignored the irregular geometry of the IVC. Similar stagnation zones were observed downstream the heads of the two filters. López et al. simulated the hemodynamic characteristics of the GreenField, Simon nitinol, VenaTech, and OptEase filters, respectively (López et al., 2018). The results further confirmed the existence of a stagnant zone downstream the filter head. Virchow’s triad indicates that three risk factors consisting of blood flow stasis, blood hypercoagulability, and endothelial damage may predispose patients to venous thrombosis (Wolberg et al., 2012). Therefore, the filter-induced regions of the blood flow stagnation and recirculation have been thought to be potentially thrombogenic, which may promote the platelet deposition and fibrin mesh network development for clot formation (Lowe, 2003; Singer et al., 2009).

For the present four cases, Filter A and Filter C have the solid cylindrical heads, which obstruct the central blood flow and result in the downstream recirculation and stagnation zone. It is worth noting that the hollow heads of Filter B and Filter D avoid the downstream recirculation, but the lengths of stagnation regions are still nearly the same as those of Filter A and Filter C (see in Figure 7), that is about 12 times *D* (see in Figure 9). Although the hollow head reduces the direct block effect and inhibit the appearance of vortex, it also increases the surface area of viscous friction, which finally does not weaken the blood flow stasis downstream the filter head.

According to the first factor of Virchow’s triad as blood flow stasis, it is unbeneficial that the solid filter head leads to both the downstream recirculation and stagnation zone (Swaminathan et al., 2006). Thus, the patterns of Filter A and Filter C still have a worrying possible complication of thrombus formation. Simulation results show that the hollow filter head is very likely to remove the vortex nearly downstream the filter head as Filter B and Filter D do. Therefore, designing an opening hole on the filter head may reduce the risk of clot accumulation around the IVC filter.

### 5.2 Acceleration effect

The viscous no-slip boundary condition requires the blood flow velocity at the filter surface to be zero. The low-speed blood flow is thus formed closely surrounding the surfaces of the filter heads and wires. The low-speed flow around the filter head is involved in the formation of the stagnation zone, which has been discussed in detail in Section 5.1. Due to the continuity of blood flow, the low-speed zones around filter wires creates an acceleration effect inside the cone and between any two wires, as shown in Figures 8B,C. Wang et al. (2020) called this acceleration mechanism the “viscous block”.

The present filter cone consisting of six long filter wires acts as a converging duct to speed the inside flow. In clinical practice, the viscous block can provide a potential benefit, washing emboli forward to the apex of filter cone to be captured. Figure 9 shows that the four different filter heads have little difference in viscous block effect.

### 5.3 Wall shear stress

As shown in Figure 10, the WSS of filter wire reaches its maximum at the upstream surface near the filter head. This agrees with the trend of blood flow velocity along the centric lines shown in Figure 9. WSS is the product of blood dynamic viscosity *μ* and flow velocity gradient (Tian et al., 2013). The no-slip boundary condition results in a large velocity gradient near the filter surface. In the flow direction, the velocity gradient inside the filter cone is increasing, and therefore the WSS at the upstream surface of filter wire also shows an increasing trend. The higher WSS on the filter surface is helpful for dissolving larger thrombus, eliminating the problem of thrombus accumulation in the short term, and has a better effect on the prevention of PE (Turitto and Hall, 1998). However, high shear rate at the filter surface may activate platelets, leading to platelet aggregation and filter thrombosis (Huang and Hellums, 1993).

The differences of four filter head patterns have little effect on the distribution of filter wire WSS. It means, for achieving the purpose of changing the filter WSS, we should do more to change the entire filter structure rather than using a new filter head. For example, Chen et al. (2017) and Selcuk et al. added the helical-shaped filter wire inside the filter cone, which obtained the increase of both the flow velocity and shear stress in the filter cone (Selimli, 2021). Additionally, the effect of a double-helical wire is more remarkable than a single-helical wire (Chen et al., 2017; Selimli, 2021). The WSS distribution on the IVC wall is also less affected by the shape of filter head. The presence of filter increases WSS on the surrounding IVC wall. It is noticeable that the peak WSS values on the IVC wall are very close for four filters with different heads, as shown in Figure 11B. However, the impact of WSS on the IVC wall induced by the IVCF is still a matter of dispute (Arzani et al., 2014), which is valuable problem worth making continuous efforts to clarify in the follow-up study.

### 5.4 Flow resistance

The flow resistance, *F*_*f*_, has two meanings: 1) it is equal to the reactive force exerted by the IVCF that impedes the blood flow; 2) it also needs to be balanced by the total force exerted by the fixed hooks at the contact locations on the IVC wall. This goes against the consensus that an ideal filter should abide by.

*F*_*f*_ consists of two components: viscous drag, *F*_*v*_ and pressure drag, *F*_*p*_. *F*_*v*_ is mainly proportional to the IVCF surface area, while *F*_*p*_ greatly depends on the geometric structure of IVCF. The patterns of filter wires for the four filters are identical. Additionally, as shown in Section 4, the shape changes in the filter head have little effect on the hemodynamic characteristics around the filter wires. Therefore, there are little differences in both *F*_*v*_ and *F*_*p*_ on the filter wires for four cases. The through hole significantly increases the surface area of the filter head. This results in *F*_*v*_ on the head of Filter D being significantly larger than those of Filter C, almost 3 times higher in value. *F*_*v*_ of Filter B is also larger than that of Filter A. The solid heads of Filter A and Filter C leads to the downstream vortex, which increases the pressure drop over the filter head. *F*_*p*_ of the filter head for Filters A and C are almost 5 times that of Filter D. However, due to the hook of filter head, *F*_*p*_ of the hollow filter head for Filter B only decreases by about 15% in comparison with that of the solid head of Filter A.

Table 3 shows that the differences of *F*_*f*_ for four cases are little. This indicates that simply changing the patten of the filter head has no obvious effect on the total flow resistance. In order to reduce *F*_*f*_, the whole structure of the IVCF should be optimized simultaneously taking into account the both contributions of *F*_*v*_ and *F*_*p*_.

## 6 Conclusion

The effects of the head pattern of IVCF have been investigated on the hemodynamic characteristics inside the IVC using CFD technique, in which the Denali filter is used as a baseline model and three variant models with different heads are also included for comparison. The simulation results show the blood flow velocity around the filter head, the WSS distribution and the flow resistance on the blood flow exerted by the IVCF, which are not discussed detailedly in previous studies. The key conclusions of this paper are as follows:

(1) The pattern of the filter head has little impact on the size of stagnation zone downstream the IVCF. The solid filter head leads to a blood flow recirculation for potential thrombosis, while the hollow head can avoid the recirculation and weaken the stagnation effect to a certain extent.
(2) For all filters, blood flow inside the filter cone and between any two filter wires can be accelerated by viscous block, improving the efficiency of clot capture. The present results show that the shape change of the IVCF head has no significant effect on this acceleration effect.
(3) Although the through hole in the IVCF head increases WSSavg of the filter head, it still has little influence on the WSS distribution on the filter wire surface and IVC wall.
(4) The structure pattern of the filter head greatly affects the flow resistance of its own. The central opening can effectively reduce the pressure drag, but increase the viscous drag. The retrieval hook can weaken the reduction of pressure drag exerted by the hollow IVCF head. However, the flow resistance of filter head only occupies a small proportion of the total resistance of IVCF for all four cases. Therefore, the flow drag reduction for an IVCF should optimize its whole structure.

## Data Availability

The original contributions presented in the study are included in the article/supplementary material, further inquiries can be directed to the corresponding authors.
